# β-Sitosterol Ameliorates Endometrium Receptivity in PCOS-Like Mice: The Mediation of Gut Microbiota

**DOI:** 10.3389/fnut.2021.667130

**Published:** 2021-06-10

**Authors:** Yanyan Yu, Ying Cao, Wenling Huang, Yanxia Liu, Ying Lu, Jiajing Zhao

**Affiliations:** ^1^Department of Gynecology, Dongfang Hospital, Beijing University of Chinese Medicine, Beijing, China; ^2^College of Traditional Chinese Medicine, North China University of Science and Technology, Tangshan, China

**Keywords:** PCOS, gut microbiota, β-sitosterol, endometrium receptivity, Bu Shen Yang Xue formula

## Abstract

**Background:** Polycystic ovary syndrome (PCOS), one of the most common endocrine diseases in women of childbearing age, has been found to be accompanied by changes in the gut microbiota. The Bu Shen Yang Xue formula (BSYXF) is a traditional Chinese medicine widely used for the treatment of PCOS. This study aimed to investigate whether the protective effects of β-sitosterol, the main active ingredient of BSYXF, on PCOS was mediated by regulating gut microbiota.

**Methods:** The presence of β-sitosterol in BSYXF was detected by liquid chromatography-mass spectrometry. The PCOS-like mouse model was induced by dehydroepiandrosterone. The fecal supernatant of β-sitosterol-treated mice was prepared for fecal microbiota transplantation (FMT). Body weight and wet weight of the uterus and ovary of the mice were recorded for organ index calculation. Hematoxylin and eosin stain was used to assess the endometrial morphology and microenvironment changes. Expression of endometrial receptivity markers cyclooxygenase-2 (COX-2), Integrin α*νβ*3, leukemia inhibitory factor (LIF), and homeobox A10 (HOXA10) in the endometrium were determined by immunohistochemistry and western blot analysis. Enzyme-linked immunosorbent assay was employed to detect the expression of follicle stimulating hormone (FSH), luteinizing hormone (LH), progesterone (P), and testosterone (T) in the serum. The diversity of gut microbiota was examined by 16S rDNA gene sequencing.

**Results:** With the treatment of β-sitosterol and β-sitosterol**-**FMT, the uterine index of PCOS-like mice increased, the ovarian index decreased, levels of COX-2, LH and T decreased, and levels of Integrin α*νβ*3, LIF, HOXA10, FSH, and P increased. Under β-sitosterol treatment, the structure of the gut microbiota in PCOS-like mice was also changed.

**Conclusion:** β-sitosterol regulates the endometrial receptivity of PCOS and harmonizes the sex hormone balance, which may be related to the changes in the structure and composition of gut microbiota, thus affecting the pathological process of PCOS.

## Introduction

Polycystic ovary syndrome (PCOS), one of the most common metabolic and endocrine disorders, affects 6–20% of women of childbearing age worldwide (1, 2). PCOS is characterized by excessive androgen secretion, low ovulation rate, and polycystic ovary (3), and is often accompanied by obesity and insulin resistance (4). Nowadays, research on women with infertility and PCOS mainly focuses on the aspects of ovulation dysfunction, sex hormones, and insulin resistance (5–7). However, insulin resistance could lead to insufficient glucose supply of endometrial cells, interfering with their growth and activity, thereby affecting endometrium receptivity (8), indicating that PCOS was closely related to endometrium receptivity.

Clinical trials have shown that PCOS is associated with decreased endometrium receptivity (9). Moreover, endocrine and metabolic abnormalities of PCOS have been found to affect the endometrium, causing endometrium disorders and leading to infertility (10). The decreased endometrium receptivity in PCOS might be caused by the noticeable imbalance of key proteins (or molecules) and signal cascades in the endometrial tissue (11). Among them, the expression of Integrin α*νβ*3, HOXA10, COX-2, and LIF in endometrium was different (9, 12, 13). Improving endometrium receptivity was reported to improve infertility in women with PCOS (14). We hypothesized that the improvement of endometrial receptivity may be a key factor in the treatment of PCOS. The level of testosterone increased in PCOS-like mice induced by DHEA (15), and testosterone was the regulator of HOXA10 (16). In addition, DHEA induces impaired decidua and endometrial receptivity in mice (17), so it is often used to simulate PCOS *in vivo*.

Intestinal microorganism disorders could cause intestinal mucosal damage and destroy the integrity of the intestinal barrier, leading to a series of diseases including PCOS (18). Clinical studies have found that changes in the gut microbiota are significantly related to the PCOS phenotype of women (19). Torres et al. (20) have conducted co-living studies with PCOS mouse model, indicating that dysbiosis of gut microbiota may be one of the causes of PCOS. A study demonstrated that *lactobacillus* and fecal microbiota transplantation (FMT) in healthy rats could treat rats with PCOS (21). In endometriosis rats treated with broad-spectrum antibiotics, metronidazole sensitive gut microbiota may promote the growth of endometrial lesions, and the feces of endometriosis rats can promote endometriosis progression (22). This suggests that intestinal microorganism plays a certain regulatory role in the development of PCOS. Gut microbiota dysregulation could lead to insulin resistance by inducing inflammation, which is closely related to endometrium receptivity (8, 23). These results suggested that the gut microbiota might influence the endometrium receptivity of PCOS through a potential mechanism.

Traditional Chinese medicine is widely used in the clinical treatment of PCOS. Studies have shown that Chinese herbal medicine has significant efficacy in promoting hormone normalization, estrus cycle recovery, insulin resistance and lipid metabolism improvement in patients with PCOS (24). The Bu Shen Yang Xue formula (BSYXF) is a traditional Chinese medicine compound, which is composed of 15 g of *Rehmannia glutinosa* (Gaertn.) DC., 15 g of *Dioscorea opposita* Thunb., 12 g of *Cervi Cornu* Colla, 15 g of *Angelica sinensis* (Oliv.) Diels, 15 g of *Dipsacus asper* Wall., 15 g of *Ligustrum lucidum* Ait., and 15 g of *Astragalus membranaceus* Moench. It has been reported that Bu Shen Huo Xue Decoction (BSHXF) has a positive effect on assisted reproduction, which was achieved by improving the morphology of rat endometrium (25). Both *Angelica sinensis* (Oliv.) Diels and *Ligustrum lucidum* Ait. in BSYXF contain β-sitosterol. β-sitosterol is one of the effective monomers in Moutan Cortex and provides an antioxidative stress effect (26). It is reasonable to assume that β-sitosterol is one of the main active ingredients in BSYXF for the treatment of PCOS. Therefore, in this study, β-sitosterol was extracted from BSYXF to investigate its influence on PCOS.

Researchers have shown that traditional Chinese medicine can improve metabolic disorders by regulating the composition and functional structure of the gut microbiota (27). For example, Guizhi Fuling Wan as a Chinese herbal medicine could control inflammation by regulating the gut microbiota, and had a certain therapeutic effect on PCOS (28). This study aimed to explore whether the effect of β-sitosterol in BSYXF on PCOS-like mice is achieved by gut microbiota, so as to provide new therapeutic targets for the treatment of PCOS.

## Materials and Methods

### Liquid Chromatography-Tandem Mass Spectrometry

The amount of β-sitosterol in BSYXF was determined by LC-MS (LC-MS-MS-8050, Shimazu, Tokyo, Japan). LC-MS analysis was performed on Waters ACQUITY UPLC T3 C18 column (100 mm × 2.1 mm, 1.7 μm). β-sitosterol (RFS-G00202004022, Chengdu Herbpurify CO., Chengdu, China) was dissolved by water to prepare the standard substance solutions. The calibration samples consisted of five nonzero concentrations of β-sitosterol, namely, 10, 20, 50, 100, and 200 ng/mL, and were used to generate the calibration curve. Approximately 1 g of BSYXF (Beijing Tcmages Pharmaceutical Co., Beijing, China) was weighed, and dissolved in 50 mL of methanol, eddied for 1 min, and centrifuged for 10 min at 10,000 rpm/min. The supernatant was diluted 100 times. The injection volume was 1 μL. The amount of β-sitosterol in each group was calculated using the standard curve of β-sitosterol.

### Animal Model of PCOS Induced by DHEA

For this study, 40 female pre-puberty C57BL/6 mice (21 days old, 17.80 ± 0.50 g) were purchased from Hunan Slack Jingda Experimental Animal Co., Ltd. (Changsha, China). All mice were randomly divided into four groups: Sham group, PCOS group, PCOS+β-sitosterol group and PCOS+β-sitosterol**-**FMT group, with 10 mice in each group. The PCOS group, PCOS+β-sitosterol group (β-sitosterol group), and PCOS+β-sitosterol**-**FMT group (β-sitosterol-FMT group) received subcutaneous injection of DHEA (20200707, OKA Biotechnology Co., Beijing, China) (6 mg/100 g body weight), 0.09 mL of sesame oil and 0.01 mL of 95% ethanol, once a day for 21 days. The Sham group was subcutaneously injected with 0.09 mL of sesame oil and 0.01 mL of 95% ethanol once a day for 21 days. The estrus cycle (proestrus, estrus, metestrus, and diestrus) was observed by a vaginal smear. When the estrus cycle of mice in the treatment group was disordered, the PCOS model was successfully established.

### β-Sitosterol Treatment and FMT

After the successful establishment of the PCOS mouse model, the β-sitosterol group was given intragastric β-sitosterol-treatment (25 mg/kg/d) for 14 consecutive days. In the β-sitosterol group, 10 g of fresh fecal samples were collected every morning after intragastric administration. The feces were stirred and mixed with 20 mL of sterile physiological saline at 37°C for 1 min, centrifuged at 1,000 g for 5 min, and the supernatant was collected. The OD value was tested at 620 nm (adjusting fecal bacterial concentration to 2 × 10^9^ CFU/mL). The supernatant was prepared for the FMT. The β-sitosterol-FMT group was given 0.2 mL of fecal supernatant from the β-sitosterol group mice by gavage for 14 consecutive days. Meanwhile, the Sham group and PCOS group were given the same amount of normal saline intragastric gavage for 14 consecutive days.

### Specimen Collection

On the last day of intragastric administration, the Sham group mice were in the metestrus stage, the PCOS group mice were constantly in the metestrus phase or diestrus phase, the β-sitosterol group and β-sitosterol-FMT group mice were in the proestrus or diestrus. All mice were weighed and then anesthetized with 2% pentobarbital sodium (30 mg/kg) for laparotomy. Abdominal aorta blood, ovaries and uterine tissues were collected. All ovaries and uterine tissues were weighed. The uterine index and ovarian index were calculated using the following formula.

$$\begin{array}{l} \text{Uterine index}=\text{ wet weight of uterus}/\text{body weight}\\ \text{Ovarian index}=\text{ wet weight of ovary}/\text{body weight} \end{array}$$

### Hematoxylin and Eosin Staining

After fixation with 4% paraformaldehyde for 4 h, the ovaries and uterine tissues of mice were dehydrated, embedded, sectioned, stained with H&E, and photographed under a microscope (BA210T, Motic, Xiamen, China) to observe the pathological structure of the ovaries and uterine tissues.

### Immunohistochemistry

The paraffin-embedded tissues were divided into four groups. The sections were dewaxed to water, antigens were heat-repaired, and endogenous enzymes were inactivated and incubated with the following primary antibodies: cyclooxygenase-2 (COX-2; ab15191, 1:1,000, Abcam, Cambridge, UK), homeobox A10 (HOXA10; ab191470, 1:5,000, Abcam, Cambridge, UK), leukemia inhibitory factor (LIF; ab138002, 1:5,000, Abcam, Cambridge, UK), and Integrin α*νβ*3 (ab179475, 1:5,000, Abcam, Cambridge, UK) at 4°C overnight. After washing with phosphate-buffered saline (PBS), the secondary antibody was incubated for 30 min, and developed with DBA, then the hematoxylin was restained, and the slices were sealed. The figures were observed and photographed under microscope (BA410T, Motic, Xiamen, China) and analyzed by image processing software (Image-Pro-Plus 6.0, Media Cybernetics, Silver Spring, USA).

### Western Blot

Total proteins were extracted from mice endometrial tissues. WB was used to detect the expression of proteins COX-2, Integrin α*νβ*3, LIF, and HOXA10. The protein was adsorbed on the PVDF membrane by gel electrophoresis and sealed with 5% skim milk solution for 2 h at room temperature. The primary antibody was incubated with COX-2 (ab15191, 1:1,000, Abcam, Cambridge, UK), HOXA10 (ab191470, 1:5,000, Abcam, Cambridge, UK), LIF (ab138002, 1:5,000, Abcam, Cambridge, UK), Integrin α*νβ*3 (ab179475, 1:5,000, Abcam, Cambridge, UK) and β-actin (66009-1-Ig, 1:5,000, Proteintech, USA) overnight at 4°C, washed three times with PBS with Tween (PBST), and secondary antibodies anti-rabbit IgG (#SA00001-2, dilution 1:6,000, Proteintech, Chicago, USA) and anti-mouse IgG (#SA00001-1, dilution 1:5,000, Proteintech, Chicago, USA) were incubated for 1.5 h at room temperature. The PBST was washed three times, and the membrane was incubated with SuperECL Plus (#K-12045-D50, Advansta, Menlo Park, USA) for 1 min. The chemiluminescence imaging system (ChemiScope 6100, Clinx, Shanghai, China) was used for scanning and imaging. β-actin was used as an internal reference for detecting relative expression levels.

### Enzyme-Linked Immunosorbent Assay

All blood samples were centrifuged at 1,000 g for 10 min and the serum was collected. Concentrations of follicle stimulating hormone (FSH), progesterone (P), luteinizing hormone (LH), and testosterone (T) in serum samples were determined by ELISA kit (CSB-E06871m, CSB-E05104m, CSB-E12770m, CSB-E05101m, CusaBio, Wuhan, China) according to the manufacturer's instructions, and all samples were repeated three times.

### 16S rDNA Sequencing

The fresh feces of all groups were collected on the last day of intragastric administration. Microbial genomic DNA was extracted from each fecal sample at 200 mg using the Fecal Genomic DNA Kit (DP328, Tiangen, Beijing, China). Moreover, 4200 TapeStation Instrument (Version 4200, Agilent Technologies, Santa Clara, USA) was used to test the quality of the extracted DNA. The whole genome of the sample was sequenced on Illumina NovaSeq platform (NovaSeq 6000, Illumina, San Diego, USA). After obtaining the original data for quality control, species composition in the samples was analyzed by comparing with the Silva-132-99 database. Data analysis was conducted using R software (Version 4.0.2, R Foundation, Vienna, Austria). First, the R software was used to generate samples or groups that had operational taxonomic unit (OTU) list, and these specific OTUs were then visualized with the help of jvenn (http://www.bioinformatics.com.cn/static/others/jvenn/example.html). Nonmetric dimensional scaling (NMDS) and analysis of similarity (ANOSIM) analysis were performed by the vegan package. The QIIME2 pipeline (2020.2) (29) was used to calculate the alpha-diversity metrics (Observe, Chao1, ACE, Shannon, and Simpon). Differential abundance at the phylum and species level was determined using the Wald test method. All plots were visualized by the package ggplot2 in R software (Version 4.0.2, R Foundation, Vienna, Austria).

### Statistical Analysis

Data are expressed as mean ± standard error of mean (SEM). GraphPad Prism 8 software (GraphPad Software, Inc., San Diego, USA) was used for statistical analysis. Comparisons among multiple groups were evaluated by one-way analysis of variance, followed by Tukey's *post-hoc* test. *P* < 0.05 was considered significant. All experiments were repeated three times. The measurement data conforms to normal distribution. The nonlinear model was used for the statistical analysis.

## Results

### Determination of β-Sitosterol in BSYXF by LC-MS

LC-MS was used to analyze the total content of β-sitosterol in Bu Shen Yang Xue formula, which was 368.636 mg/kg (Supplementary Figure 1).

### Effects of β-Sitosterol on Ovaries and Uterus in PCOS-Like Mice

**Figure 1:**
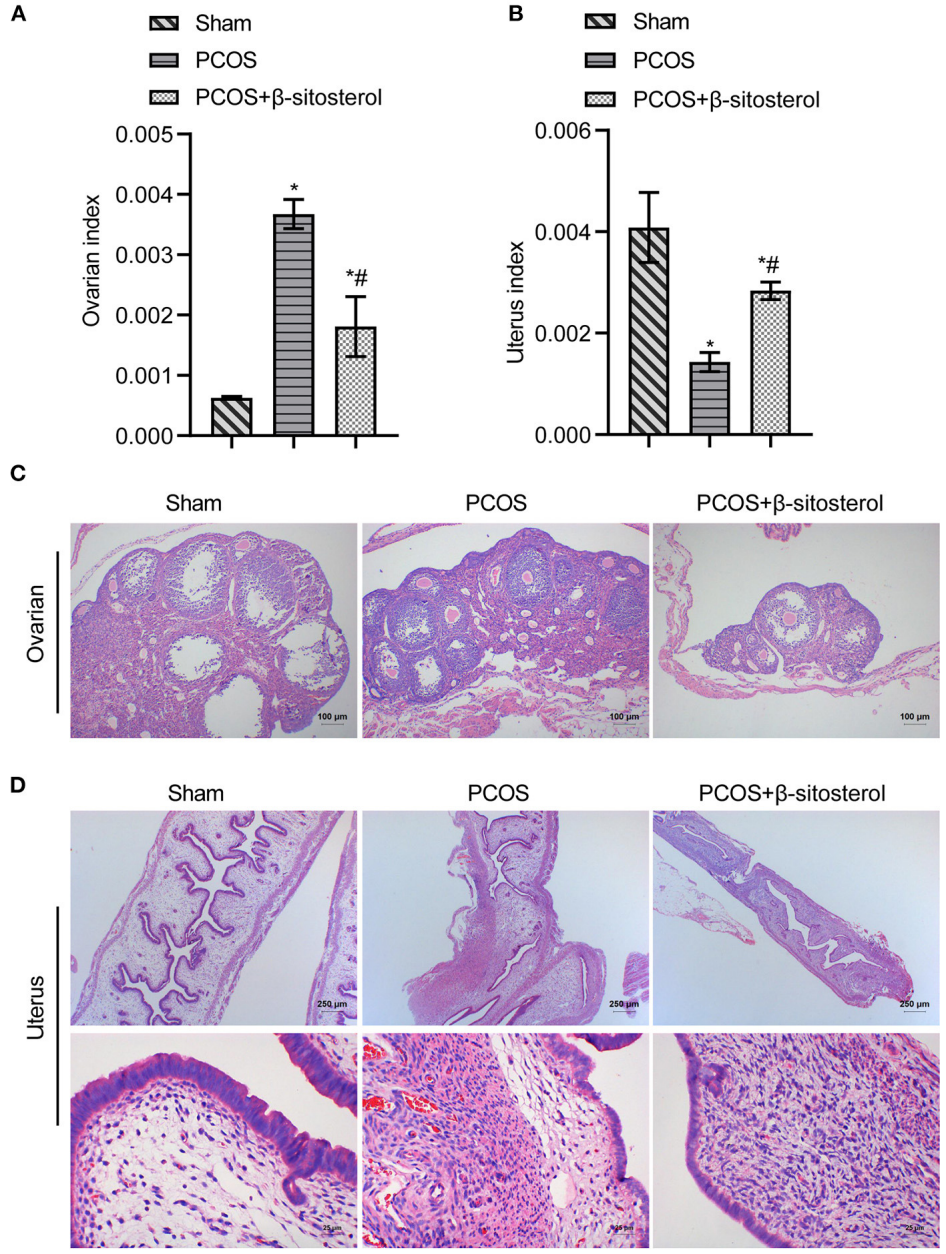
Effect of β-sitosterol on ovaries and uterus in PCOS-like mice. **(A)** Ovarian index. **(B)** Uterine index. **(C)** Hematoxylin and eosin (H&E) staining was performed to observe pathological changes of ovarian tissue in each group (magnification, 100×; scale bar = 100 μm). **(D)** H&E staining showed pathological changes of uterine tissue in each group. Upper images are magnified 40-fold (scale bar = 250 μm), and local magnification (underneath) is magnified 400-fold (Scale bar = 25 μm). Data are presented as mean ± SEM. *P < 0.05 vs. Sham. ^#^*P* < 0.05 vs. PCOS. PCOS, polycystic ovary syndrome.

To investigate the curative effect of β-sitosterol on PCOS-like mice, we first evaluated the physiological state of mice in different groups. The ovarian index of the PCOS group was significantly higher than that of the Sham group, while the uterine index of the PCOS group was lower than that of the Sham group. Both indices were notably reversed after β-sitosterol treatment (Figures 1A,B). After β-sitosterol treatment, results of H&E staining showed that excessive ovarian vesicles were reduced and absent granulosa cell layers were evidently increased in PCOS-like mice (Figure 1C). From uterine H&E staining, the average thickness of the endometrium of mice in the PCOS+β-sitosterol group was thicker than that in the PCOS group (Figure 1D). These results implied that β-sitosterol is capable of improving the uterine and ovary status of PCOS-like mice.

### Effects of β-Sitosterol on the Expression of Endometrium Receptivity Markers and Related Hormones in PCOS-Like Mice

**Figure 2:**
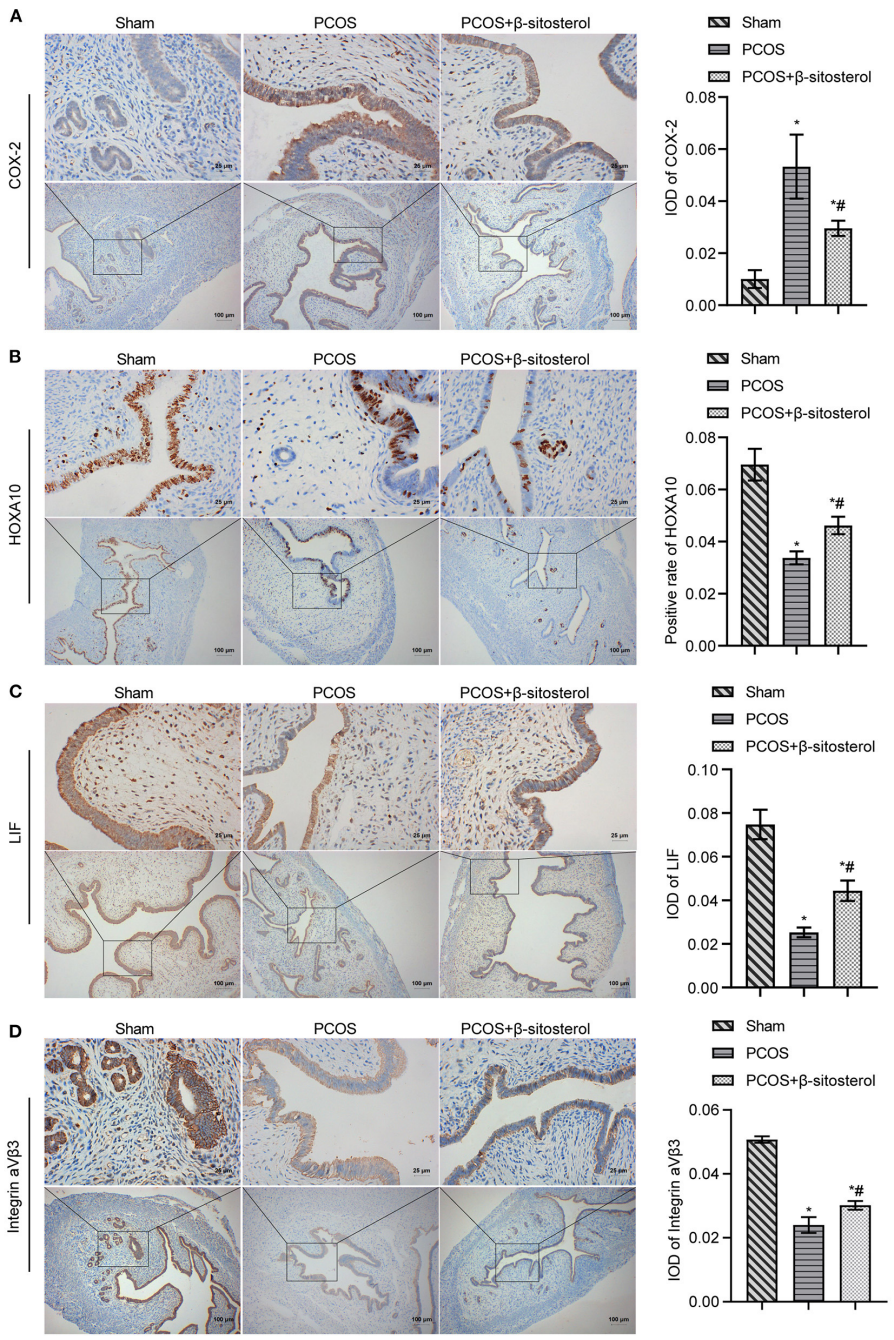
Effect of β-sitosterol on the expression of endometrium receptivity markers and related hormones in PCOS-like mice. **(A–D)** Expressions of COX-2, HOXA10, LIF, and Integrin α*νβ*3 in the endometrium of mice in each group detected by IHC. Upper images are magnified 400-fold (scale bar = 25 μm) and lower images are magnified 100-fold (scale bar = 100 μm). Data are presented as mean ± SEM. *P < 0.05 vs. Sham. ^#^*P* < 0.05 vs. PCOS. PCOS, polycystic ovary syndrome; COX-2, cyclooxygenase-2; HOXA10, homeobox A10; LIF, leukemia inhibitory factor; ELISA, enzyme-linked immunosorbent assay.

**Figure 3:**
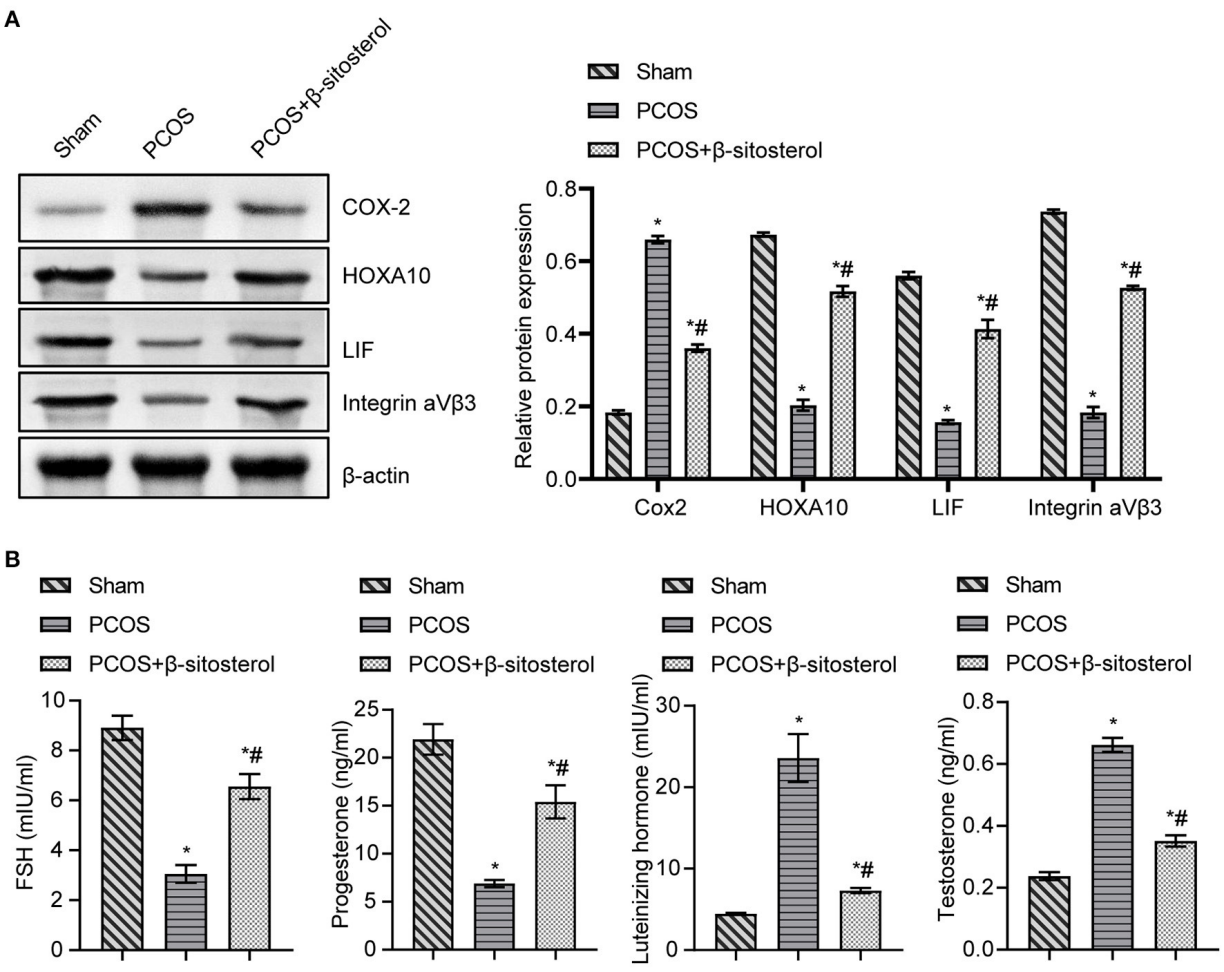
Effect of β-sitosterol on the expression of endometrium receptivity markers and related hormones in PCOS-like mice. **(A)** Expressions of COX-2, HOXA10, LIF, and Integrin α*νβ*3 in the endometrium of mice in each group were detected by western blot. **(B)** Levels of FSH, P, LH, and T in the serum of mice in each group were detected by ELISA. Data are presented as mean ± SEM. *P < 0.05 vs. Sham. ^#^*P* < 0.05 vs. PCOS. FSH, follicle-stimulating hormone; P, progesterone; LH, luteinizing hormone; T, testosterone; PCOS, polycystic ovary syndrome.

To further verify the effect of β-sitosterol on the endometrium receptivity of PCOS-like mice, the expressions of COX-2, Integrin α*νβ*3, LIF and HOXA10 in the endometrium of each group was detected by IHC and then WB. IHC results showed that the expression of COX-2 was markedly downregulated after β-sitosterol treatment in PCOS-like mice (Figure 2A). Meanwhile, β-sitosterol effectively inhibited the excessive decrease in expressions of integrins α*νβ*3, LIF, and HOXA10 in the endometrium of PCOS-like mice (Figures 2B–D). Results of WB were consistent (Figure 3A). Then, ELISA was used to detect the serum sex hormone levels of mice in each group. β-sitosterol treatment was observed to increase FSH and P levels in PCOS-like mice. By contrast, serum LH and T levels were significantly reduced in PCOS-like mice treated with β-sitosterol (Figure 3B). These results indicated that β-sitosterol has a positive effect on endometrial receptivity and on the sex hormone balance of PCOS-like mice.

### Effects of β-Sitosterol on the Composition of Gut Microbiota in PCOS-Like Mice

**Figure 4:**
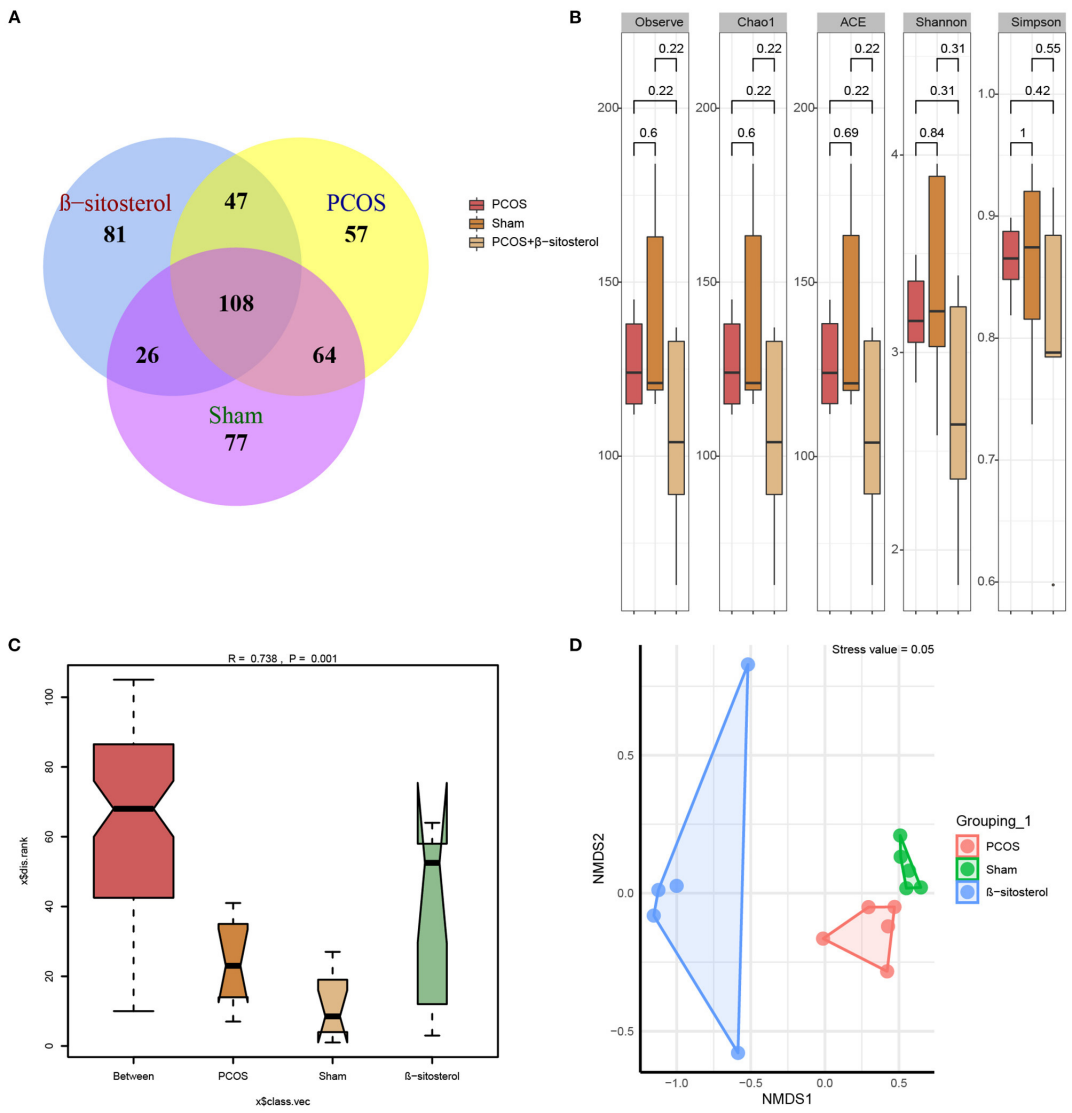
Effects of β-sitosterol on the composition of gut microbiota in PCOS-like mice. **(A)** Venn diagrams demonstrated the results of operational taxonomic units in groups. **(B)** Changes in Observe, Chao1, ACE, Shannon, and Simpon indices. **(C)** Analysis of similarities showed differences in groups. **(D)** Nonmetric multidimensional scaling analysis showed differences in groups. PCOS, polycystic ovary syndrome; FSH, follicle-stimulating hormone; P, progesterone; LH, luteinizing hormone; T, testosterone.

Studies have shown that abnormal changes in gut microbiota was implicated in PCOS (21). Therefore, we hypothesized that regulating gut microbiota may play a role in the improvement effect of β-sitosterol on PCOS. Thereafter, the 16S rDNA gene sequencing was used to analyze the gut microbiota diversity. Based on the species annotation analysis of OTU, the Venn plot showed that the unique OTUs in the Sham group, PCOS group, and β-sitosterol group were 77, 57, and 81, respectively (Figure 4A). Moreover, alpha diversity was statistically analyzed. Unexpectedly, an undifferentiated distinction in microbial diversity as displayed by the observed index, Chao1 index, Ace index, Shannon index, and Simpon index, was observed in the three groups (Figure 4B). ANOSIM is a nonparametric test to check whether the differences between groups are significantly greater than the differences within groups, and therefore whether the grouping is meaningful. ANOSIM showed that this observation (*R* = 0.738, *P* = 0.001) was significant in the study groups (Figure 4C). Uniformly, NMDS analysis results showed that all groups of samples were separated clearly (Figure 4D). Therefore, it is reasonable to infer that β-sitosterol treatment may exert a protective effect by altering the species and structure of specific gut microbiota.

### Effects of β-Sitosterol on the Abundance of Specific Microbiota in PCOS-Like Mice

**Figure 5:**
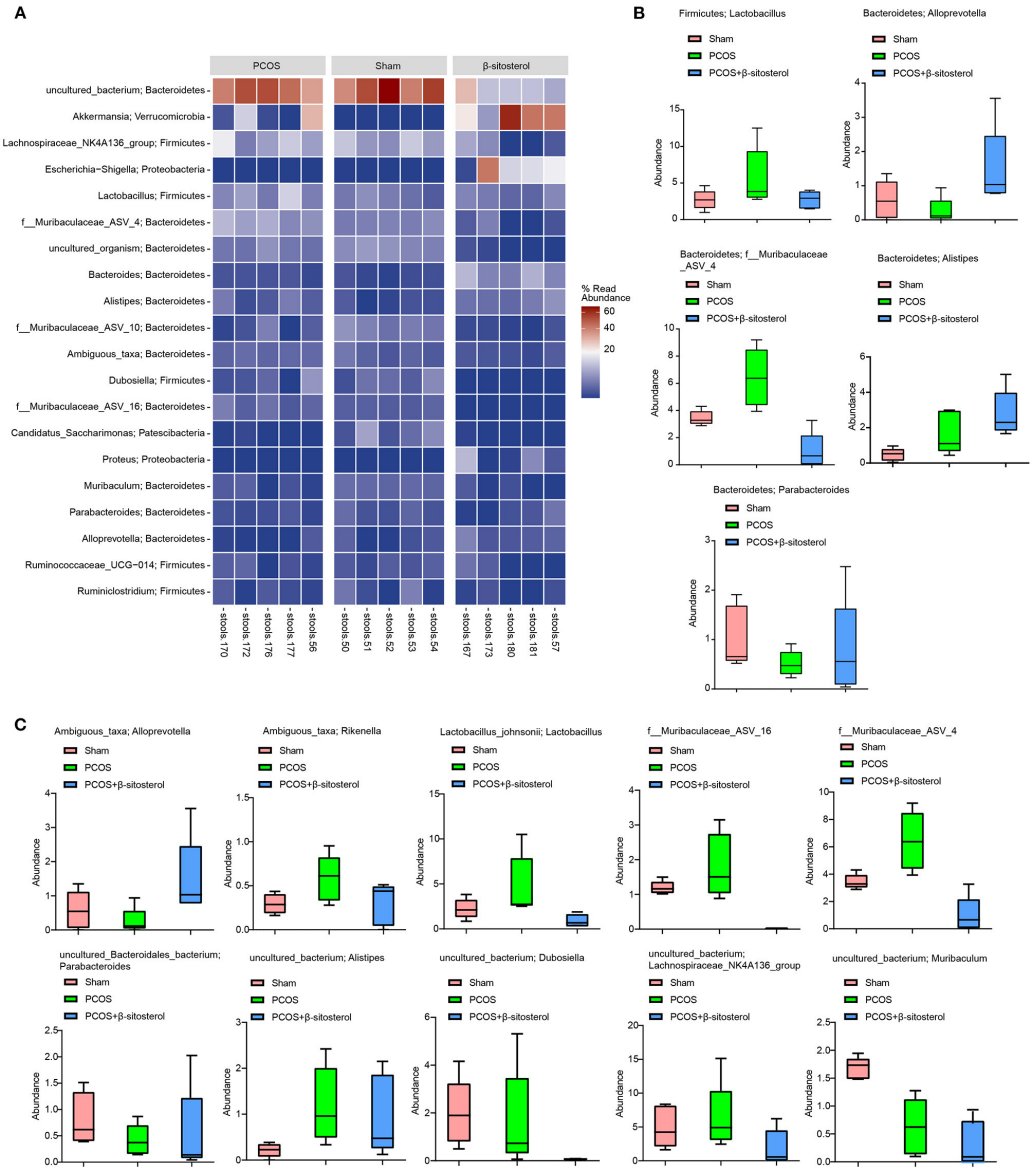
Effects of β-sitosterol on the abundance of specific microbiota in PCOS-like mice. **(A)** Heat map showed that the abundances of the first 20 species were differentially expressed in groups. **(B)** Relative abundance of the five bacterial categories at the phylum level. Top five bacteria: *Firmicutes-Lactobacillus, Bacteroidetes-Alloprevotella, Bacteroidetes-Parabacteroides, Bacteroidetes-f_Muribaculaceae_ASV_4*, and *Bacteroidetes-Alistipes*. **(C)** Relative abundance of the top 10 bacterial categories at the species level. Top 10 bacteria: *Ambiguous_taxa-Alloprevotella, Ambiguous_taxa-Rikenella, Lactobacillus_johnsonii-Lactobacillus, f_Muribaculaceae_ASV_16, f_Muribaculaceae_ASV_4, uncultured_Bacteroidales_bacterium-Parabacteroides, uncultured_bacterium-Alistipes, uncultured_bacterium-Dubosiella, uncultured_bacterium-Lachnospiraceae_NK4A-136_group*, and *uncultured_bacterium-Muribaculum*. PCOS, polycystic ovary syndrome.

To further explore the differences in the relative abundance of bacterial taxa, the relative abundance of the top 20 bacterial taxa in the three groups was assessed by the cluster heat map (Figure 5A). Moreover, differences in the relative abundance of the gut microbiota in each group at the phylum level were statistically analyzed. The top five different categories of bacteria in the phylum level were analyzed emphatically. Abundances of *Firmicutes-Lactobacillus, Bacteroidetes-f_Muribaculaceae_ASV_4*, and *Bacteroidetes-Alistipes* showed an upward trend in PCOS group, while in β-sitosterol group the change in these taxa was reversed. Inversely, after treatment with β-sitosterol, the decrease in *Firmicutes-Lactobacillus, Bacteroidetes-alloprevotella, Bacteroidetes-parabacteroides*, and *Bacteroidetes-f_Muribaculaceae_ASV_4* in PCOS-like mice was improved (Figure 5B). Furthermore, the abundance of the top 10 bacteria at the species level was analyzed. Although the abundances of *Ambiguous_taxa-Rikenella, Lactobacillus_johnsonii-Lactobacillus, f_Muribaculaceae_ASV_16, f_Muribaculaceae_ASV_4, uncultured_bacterium-Alistipes, uncultured_bacterium-Dubosiella*, and *uncultured_bacterium-Lachnospiraceae_NK4A136_group* demonstrated an upward trend in the PCOS group compared with that in the Sham group, all these taxa abundances were reduced to some extent by β-sitosterol. In addition, the relative abundance of *Ambiguous_taxa-Alloprevotella, uncultured_Bacteroidales_bacterium-Parabacteroides*, and *uncultured_bacterium-Muribaculum* in the β-sitosterol group was counter to that in PCOS-like mice, which showed a partial increasing trend (Figure 5C). Although certain microbial strains such as *Firmicutes-Lactobacillus and Ambiguous_taxa-Alloprevotella* showed no significant difference among all groups, the variation tendency was still observed. These results indicated that β-sitosterol has the potential to change the intestinal microflora structure of PCOS-like mice and restore it to near normal levels.

### Effects of β-Sitosterol-FMT on Ovaries and Uterus in PCOS-Like Mice

**Figure 6:**
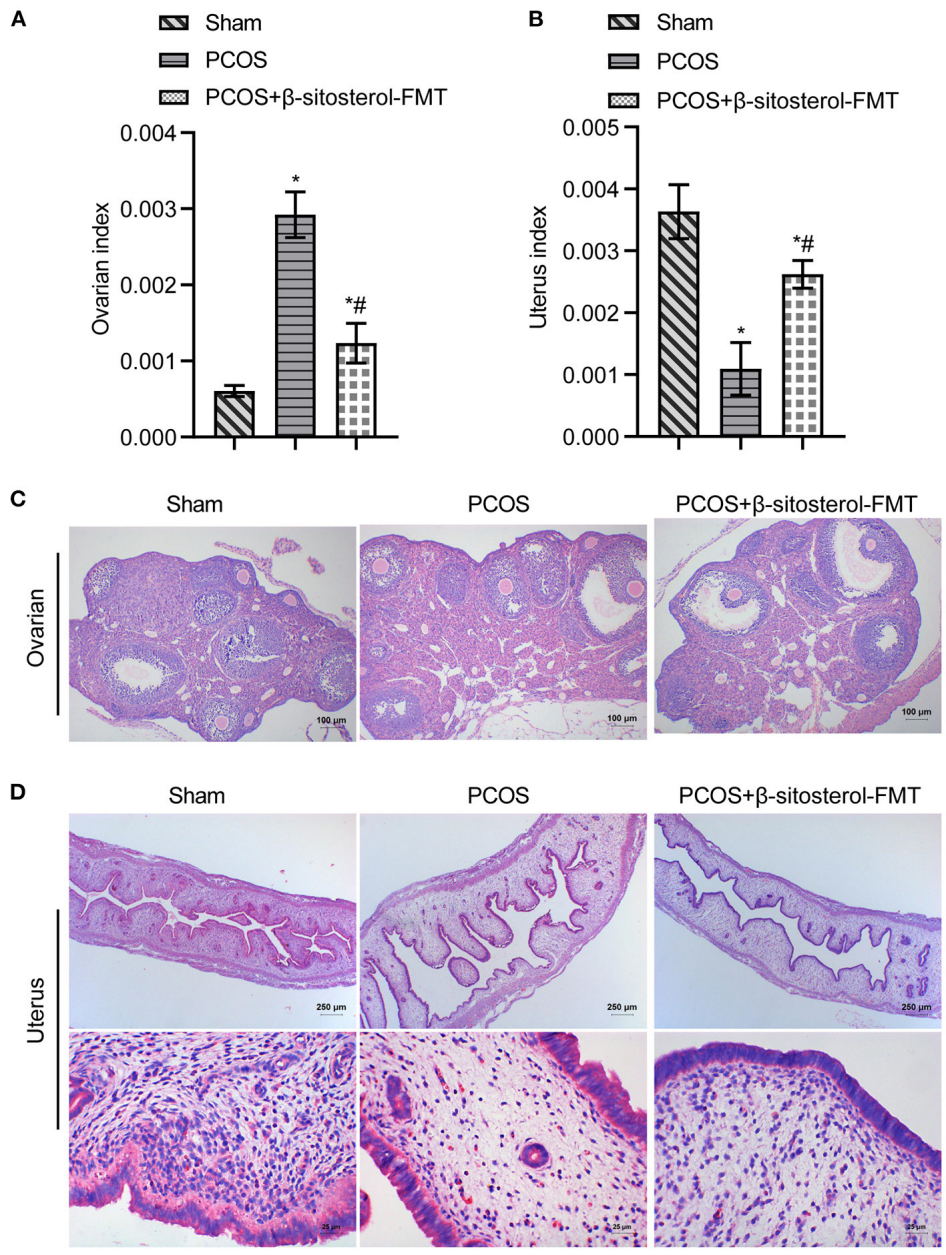
Effects of β-sitosterol-FMT on ovaries and uterus in mice with PCOS. **(A)** Ovarian index. **(B)** Uterine index. **(C)** Hematoxylin and eosin (H&E) staining showed pathological changes of ovarian tissue in each group. Images are magnified 100-fold (scale bar = 100 μm). **(D)** HE staining showed pathological changes of uterine tissue in each group. Upper images are magnified 40-fold (scale bar = 250 μm), and underneath images are magnified 400-fold (scale bar = 25 μm). Data are presented as mean ± SEM. *P < 0.05 vs. Sham. ^#^*P* < 0.05 vs. PCOS. PCOS, polycystic ovary syndrome.

Furthermore, feces of mice from the β-sitosterol treatment group were transplanted into PCOS-like mice to confirm that β-sitosterol plays a positive role in PCOS through the intestinal flora. Compared with the PCOS group, the uterine index of mice in the β-sitosterol-FMT group showed a marked increase, compared with that of the ovarian index (Figures 6A,B). H&E staining results showed that, after β-sitosterol-FMT treatment, cystic follicles decreased and granulosa cell layer increased in PCOS-like mice (Figure 6C). H&E staining of the endometrium showed that the average thickness of the endometrium of mice in the β-sitosterol-FMT group increased compared with the mice in the PCOS group (Figure 6D). Therefore, the gut microbiota may be a potential pathway for β-sitosterol to ameliorate PCOS.

### Effect of β-Sitosterol-FMT on the Expression of Endometrium Receptivity Markers and Related Hormones in PCOS-Like Mice

**Figure 7:**
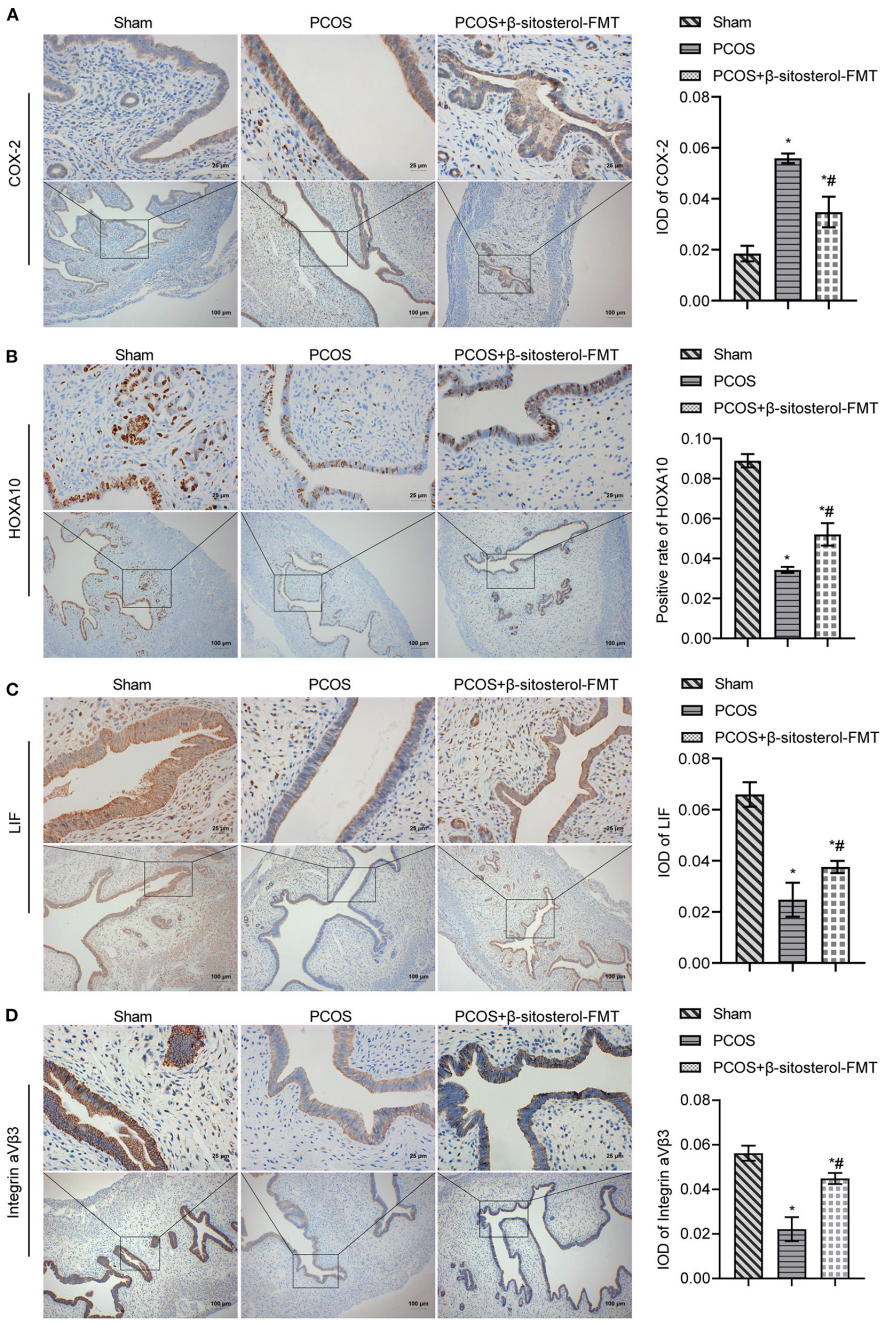
Effect of β-sitosterol-FMT on the expressions of endometrium receptivity markers and related hormones in mice with PCOS. **(A–D)** Expressions of COX-2, HOXA10, LIF, and Integrin α*νβ*3 in the endometrium of mice in each group were detected by IHC. Upper images are magnified 400-fold (scale bar = 25 μm), and underneath images are magnified 100-fold (scale bar = 100 μm). Data are presented as mean ± SEM. *P < 0.05 vs. Sham. ^#^*P* < 0.05 vs. PCOS. PCOS, polycystic ovary syndrome; COX-2, cyclooxygenase-2; HOXA10, homeobox A10; LIF, leukemia inhibitory factor; ELISA, enzyme-linked immunosorbent assay.

**Figure 8:**
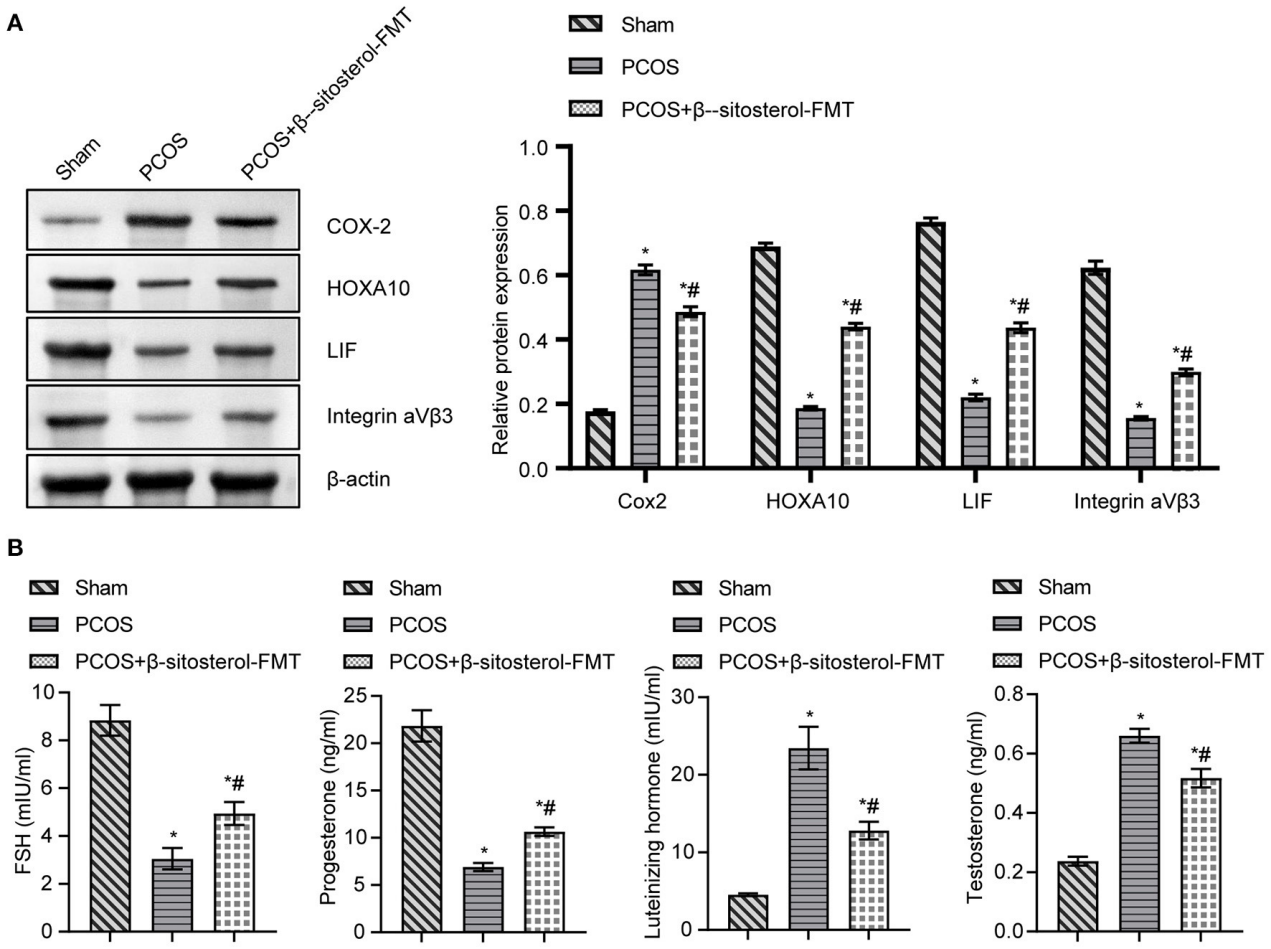
Effect of β-sitosterol-FMT on the expressions of endometrium receptivity markers and related hormones in mice with PCOS. **(A)** Expressions of COX-2, HOXA10, LIF, and Integrin α*νβ*3 in the endometrium of mice in each group were detected by western blot. **(B)** Levels of FSH, P, LH, and T in the serum of mice in each group were detected by ELISA. Data are presented as mean ± SEM. *P < 0.05 vs. Sham. ^#^*P* < 0.05 vs. PCOS. PCOS, polycystic ovary syndrome; COX-2, cyclooxygenase-2; HOXA10, homeobox A10; LIF, leukemia inhibitory factor; ELISA, enzyme-linked immunosorbent assay.

To further investigate the effect of β-sitosterol-FMT on the endometrium receptivity of PCOS-like mice, the expressions of endometrial receptivity marker proteins in each group was detected by IHC and WB. As a result, the expression of COX-2 was significantly decreased (Figure 7A) and the expressions of Integrin α*νβ*3, LIF, and HOXA10 were significantly increased in the β-sitosterol-FMT group compared with that in the PCOS group (Figures 7B–D). As shown in Figure 8A, WB detection results were consistent with IHC results, which demonstrated that β-sitosterol-FMT improves the endometrium receptivity of PCOS-like mice. According to ELISA results (Figure 8B), the levels of FSH and P in the β-sitosterol-FMT group mice were significantly increased compared with those in the PCOS group. By contrast, when PCOS-like mice were treated with β-sitosterol-FMT, the levels of LH and T were significantly lower than those in the PCOS group. The above results manifested that β-sitosterol-FMT treatment also have a positive effects on the sex hormone balance of PCOS-like mice.

## Discussion

In our study, we established a mouse model of PCOS induced by DHEA, and found that β-sitosterol improved endometrial receptivity and balanced sex hormone levels in mice with PCOS. In addition, β-sitosterol can improve the composition of gut microbiota in PCOS-like mice. The gut microbiota composition of PCOS-like mice was improved after β-sitosterol treatment. The feces of β-sitosterol-treated mice were transplanted into PCOS, demonstrating that β-sitosterol may have a positive effect on PCOS-like mice by regulating gut microbiota. Our study suggested that β-sitosterol is capable of altering the gut microbiota imbalance in the pathogenesis of PCOS and of improving the development process of PCOS.

Many literature have reported that DHEA induced rodent models with remarkable characteristics of polycystic ovary syndrome (30–33). The reversal of FSH/LH ratio is an important clinical feature of PCOS (34). In addition, it has been reported that LH level increased (15) and FSH expression level decreased (35) in PCOS model induced by DHEA. Therefore, the DHEA induced PCOS model is a feasible method. However, it has been reported that there may be no difference in LH and/or FSH levels between DHEA-induced PCOS model and control group, which may be the result of the difference in model establishment. In addition, DHEA treatment can significantly increase the number of cystic follicles and the thickness of membrane cell layer in mice, and significantly reduce the number of corpus luteum and dominant follicles, indicating that DHEA can induce the formation of PCOS in mice (36). This is consistent with our research.

PCOS was an important cause of female infertility, which might cause various serious complications (37, 38). Thus, studying effective treatment of PCOS is an urgent issue to improve the physical condition of patients with PCOS. Previous studies have confirmed that β-sitosterol was very effective in treating anti-inflammatory (39), antioxidative stress (40) and antitumor (41). In this study, β-sitosterol treatment significantly improved the uterine and ovary structure in PCOS group, we therefore hypothesize from these observations that β-sitosterol treatment may have some effect on the improvement of PCOS. Studies have shown that decreased endometrium receptivity is an important indicator of infertility in patients with PCOS (42). Endometrial receptivity was reduced when PCOS occurs, and relevant biomarkers are abnormally expressed (9). We first investigated the therapeutic effect of β-sitosterol on the endometrial receptivity of PCOS-like mice. The observation of β-sitosterol evidently reversed a low expression of Integrin α*νβ*3, LIF, and HOXA10 and a high expression of COX-2 in the PCOS group, suggests β-sitosterol in PCOS group altered these abnormal expressions of markers in the endometrium. A disorder of sex hormone secretion was another cause of PCOS (43). In our study, β-sitosterol distinctly reduced the production of serum FSH and P in PCOS-like mice and promoted the production of LH and T. It is tempting to speculate from these observations that β-sitosterol could not only modulate endometrial receptivity, but also coordinate sex hormone balance in PCOS-like mice.

During the past decades, the regulatory roles of gut microbiota on various diseases, including PCOS, have gained increasing attention (44, 45). β-sitosterol could improve rumen fermentation in sheep by reducing microbial community and metabolic disorders induced by high grain feed (46). Therefore, we have reason to suspect that the protective effect of β-sitosterol on PCOS may be related to gut microbiota. In this study, 16S rDNA sequencing of gut microbiota showed that β-sitosterol had a regulatory effect on gut microbiota of mice in the PCOS group. Nevertheless, β- sitosterol showed no significant effect on the alpha diversity of PCOS-like mice. In a study that explored the composition of the gut flora in women with PCOS, no change was found in the alpha-diversity of the gut flora between patients with PCOS and people with good health status (47). We speculate that this may be due to the small sample size of the intestinal microbiome. To our delight, we found significant differences in beta diversity among various treatments. Moreover, the relative abundance of some bacterial communities changed significantly with the addition of β-sitosterol. A study found that *bacteroidetes* in PCOS has a lower relative abundance (48). Our results are consistent with such finding. In addition, we found that *Ambiguous_taxa-Alloprevotella* and *Parabacteroides* decreased in the intestinal tract of PCOS-like mice. After treatment with β-sitosterol, the structure of the gut microbiota in the PCOS group was significantly changed. Zhu et al. (28) found that the relative abundance of *Alloprevotella* was decreased significantly in PCOS. However, *Bacteroidete-Alloprevotella* and *Ambiguous_taxa-Alloprevotella* were upregulated by β-sitosterol administration, whereas *Firmicutes-Lactobacillus, Bacteroidetes-f_Muribaculaceae_ASV_4*, and *f_Muribaculaceae_ASV_16* were downregulated. Therefore, it is reasonable to speculate that β-sitosterol affects PCOS by changing the structure of the gut microbiota.

FMT was an innovative method for the treatment of PCOS. Gut microbiota disorders could be restored by FMT from healthy donors to recipients (49). FMT of healthy rats could improve the estrus cycle and ovarian disorder of PCOS rats (21). In our study, FMT in β-sitosterol treated mice restored endometrium receptivity of PCOS-like mice. It also decreased the levels of FSH and P and increased the levels of LH and T. β-sitosterol-FMT assisted in the treatment of PCOS-like mice. It is tempting to speculate from these observations that β-sitosterol has the ability to harmonize gut microbiota homeostasis in PCOS-like mice.

Our results indicate that the therapeutic effect of β-sitosterol on PCOS-like mice is at least partially mediated by the improvement of intestinal microbiota composition, suggesting that β-sitosterol may be an effective treatment for PCOS. However, it is unclear how β-sitosterol in the gut microbiota improves endometrial receptivity of PCOS. Given the small sample sizes no significant difference was found in the relative abundance of some intestinal microorganisms among the experimental groups. The exact cellular and molecular mechanisms by which β-sitosterol change the composition of gut microbes these changes is also unclear. In our next experiment, we will collect more samples for more precise experiments. In addition, we will further explore the influence of intestinal flora on PCOS in combination with clinical and animal experiments, as well as the ways through which β-sitosterol influences intestinal flora, so as to exert its alleviating effect on PCOS.

## Conclusion

We found that β-sitosterol can regulate endometrial receptivity and sex hormone balance in PCOS-like mice, which may be related to its regulation effect on gut microbiota. At the same time, β-sitosterol-treated mice feces transplanted into PCOS-like mice, also contributed to the improvement of PCOS. Results suggested that β-sitosterol has a good clinical application prospect in the treatment of PCOS.

## Data Availability Statement

The datasets presented in this study can be found in online repositories. The names of the repository/repositories and accession number(s) can be found below: https://www.ncbi.nlm.nih.gov (PRJNA703774).

## Ethics Statement

The animal study was reviewed and approved by the Ethics Committee of the Beijing University of Chinese Medicine Animal Care and Use Committee.

## Author Contributions

YY and YC performed the experiment and analyzed the data. JZ, YiL, and YaL performed the experiment. WH, YC, and YY guided the experiment, reviewed, and edited the manuscript. All authors contributed to the article and approved the submitted version.

## Conflict of Interest

The authors declare that the research was conducted in the absence of any commercial or financial relationships that could be construed as a potential conflict of interest.

## Supplementary Material

Supplementary Figure 1 Determination of β-sitosterol in Bu Shen Yang Xue formula by liquid chromatography-tandem mass spectrometry.
