# Supplementary figures and images for: β-Sitosterol Ameliorates Endometrium Receptivity in PCOS-Like Mice: The Mediation of Gut Microbiota
Source: Front Nutr. 2021 Jun 10;8:667130. doi: 10.3389/fnut.2021.667130 (PMC8224531; doi:10.3389/fnut.2021.667130)

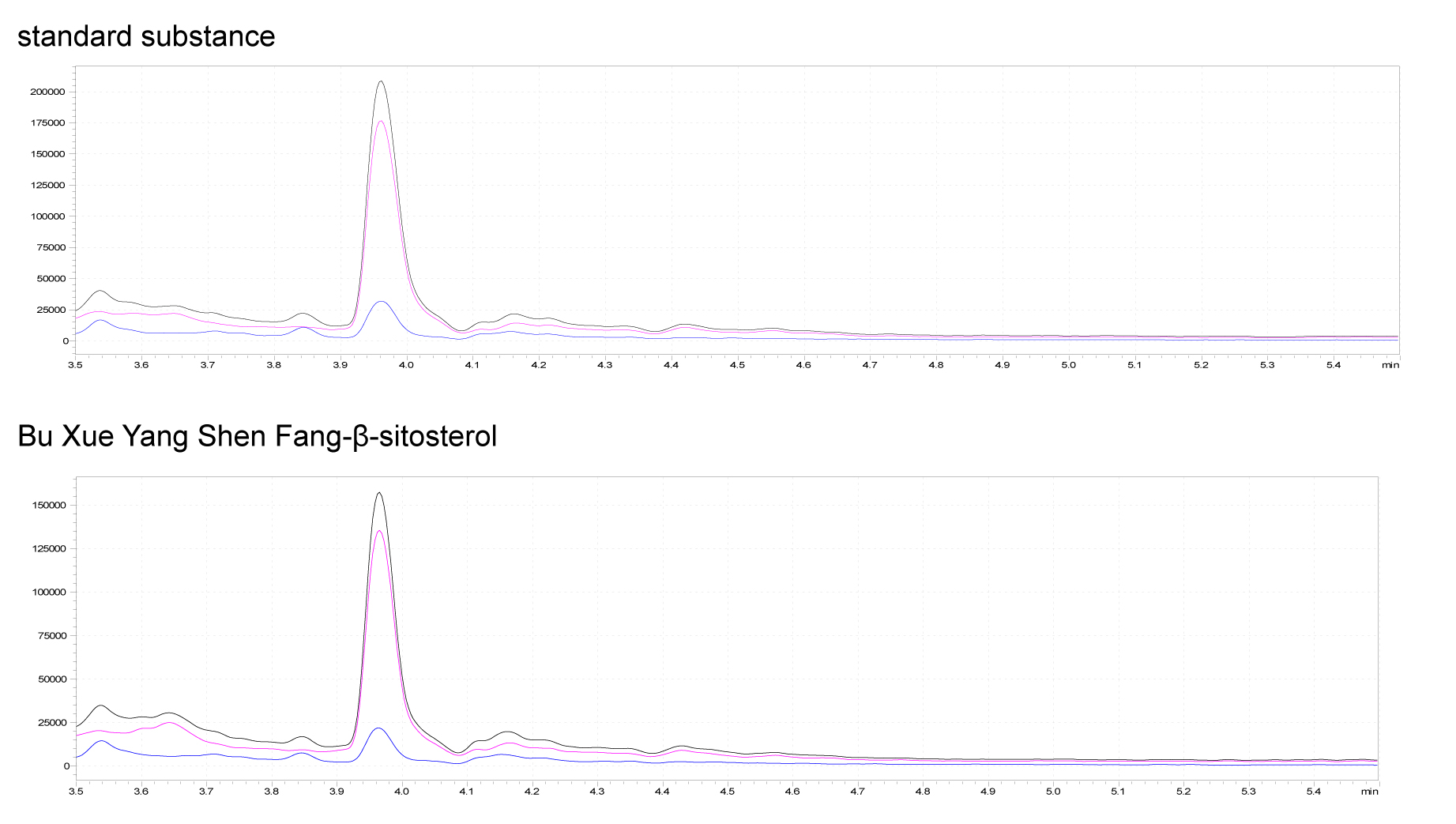

Supplement: Supplementary Figure 1 — Determination of β-sitosterol in Bu Shen Yang Xue formula by liquid chromatography-tandem mass spectrometry. [file Image_1.JPEG]
